# Supplementary material for: Assessment of Lipid Peroxidation Products in Adult Formulas: GC-MS Determination of Carbonyl and Volatile Compounds Under Different Storage Conditions
Source: Foods. 2024 Nov 23;13(23):3752. doi: 10.3390/foods13233752 (PMC11640357; doi:10.3390/foods13233752)
Supplement: Supplementary file 1 [file foods-13-03752-s001.zip › foods-3317543-supplementary.pdf]

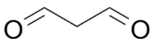

Malondialdehyde
($C_3H_4O_2$)
72.06 g/mol

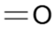

Formaldehyde
(CH_2O)
30.03 g/mol

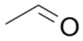

Acetaldehyde
(C_2H_4O)
44.05 g/mol

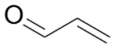

Acrolein
(C_3H_4O)
56.06 g/mol

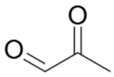

Methylglyoxal
($C_3H_4O_2$)
72.06 g/mol

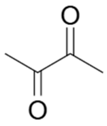

Diacetyl
($C_4H_6O_2$)
86.09 g/mol

Figure S1. Structural formula, molecular formula, and molecular weight of target carbonyl compounds.

Table S1. Retention time, quantifier and qualifier ions for the US-DLLME-GC-MS method.

Analyte	Retention Time min	Quantifier ion m/z	Qualifier ions m/z	m/z
Malondialdehyde	4.03	234	204	2017
Formaldehyde	4.03	210	180	211
Acetaldehyde	4.97	224	225	180
Acetaldehyde-d4*	4.81	228	227	229
Acetone-d6*	5.44	244	243	183
Acrolein	5.50	236	189	201
Methylglyoxal	6.07	252	253	236
Diacetyl	6.18	266	267	181

*used as internal standard

Table S2. Analytical methods for malondialdehyde, acrolein, and α-dicarbonyl compounds in infant and adult formulae, milk, and dairy products.

Sample	Analytes	Sample preparation	Derivatization	Apparatus	LOD ng g^{-1}	LOQ ng g^{-1}	r^2	Recovery %	RSD %	Ref
Infant formula	MDA	LLE	TBA	HPLC-UV	NR	NR	0.9827	40-80	<13.7	[13]
Infant formula	MDA	Dilution	TBARS method	HPLC-UV	9.7	NR	0.9986	70-78	4.6	[14]
Infant formula	MGO	MWAH		HPLC-MS	4.5	18.1	0.996	103	<8.1	[15]
Milk	FCHO	DLLME	ACAC	UV	$1x10^5$	$5x10^5$	NR	91-103	<3.1	[16]
Baby food	MGO, DA	SLE	*o*-PDA	HPLC-MS	2.7-6.0	9.2-20.1	NR	96-103	<5	[24]
Milk and diary	MGO, DA	Protein precipitation	*o*-PDA	HPLC-MS/MS	5-7	18-23	>0.99	85-83	<8.4	[18]
Infant formula	MDA, ACRL, MGO, DA	GDME	*o*-PDA	HPLC-UV	30-300	100-1000	>0.9993	93-105	<4.8	[19]
Infant formula	MDA, FCHO, ACE, MGO, DA	SPME	PFPH	GC-MS	15-50	50-150	>0.9990	84-111	<14.7	[20]
Adult formula	MDA, FCHO, ACE, ACRL, MGO, DA	DLLME	DNPH	GC-MS	8-89	61-671	>0.9990	98-102	<6.9	This work

ACAC, acetylacetone in an ammonium acetate (2.0% v/v); DNPH, 2,4-dinitrophenylhydrazine; LLE, liquid-liquid extraction; MWAH, microwave-assisted hydrolysis; NR, not reported; o-PDA, o-phenylene diamine; PFPH, pentafluorophenyl hydrazine; RSD, relative standard deviation; SLE, solid-liquid extraction; TBA, 2-thiobarbituric acid; TBARS, thiobarbituric acid reactive substances.

Table S3. Volatile compounds identified in adult formulas.

Compound	Retention Time (min)	Molecular Weight (g/mol)	Molecular Formula	CAS Number
**Aldehydes**				
Hexanal	3.961	100.16	$C_6H_{12}O$	66-25-1
2-Ethylbutenal	4.911	98.15	$C_6H_{10}O$	8/1/4786
Heptanal	7.413	114.19	$C_7H_{14}O$	111-71-7
Benzaldehyde	9.67	106.12	C_7H_6O	100-52-7
Octanal	11.507	128.21	$C_8H_{16}O$	124-13-0
2-Heptenal	9.59	110.18	$C_7H_{12}O$	18829-55-5
trans-2-Octenal	13.83	126.2	$C_8H_{14}O$	2548-87-0
Nonanal	15.634	142.24	$C_9H_{18}O$	124-19-6
2-Hydroxybenzaldehyde	16.292	122.12	$C_7H_6O_2$	90-02-8
Decanal	19.403	156.26	$C_{10}H_{20}O$	112-31-2
2-Undecenal	24.853	168.29	$C_{11}H_{20}O$	2463-77-6
**Ketones**				
2-Heptanone	7.023	114.19	$C_7H_{14}O$	110-43-0
6-Methyl-5-hepten-2-one	10.874	126.2	$C_8H_{14}O$	110-93-0
3-Octen-2-one	13.01	126.2	$C_8H_{14}O$	1669-45-8
2-Nonanone	15.146	128.21	$C_9H_{18}O$	821-55-6
3,5-Octadien-2-one	15.1385	124.18	$C_8H_{12}O$	3/5/4313
3-Nonen-2-one	16.949	126.2	$C_9H_{16}O$	18829-56-6
Cyclodecanone	21.515	154.25	$C_{10}H_{18}O$	1502-06-3

6-Undecanone	21.767	170.29	$C_{11}H_{22}O$	13161-21-6
2-Decanone	18.94	142.24	$C_{10}H_{20}O$	693-54-9
2-Undecanone	22.522	156.26	$C_{11}H_{22}O$	112-12-9
**Alcohols**				
1-Pentanol	4.676	88.15	$C_5H_{12}O$	71-41-0
1-Hexanol	6.138	102.17	$C_6H_{14}O$	111-27-3
1-Heptanol	10.272	116.2	$C_7H_{16}O$	111-70-6
1-Octen-3-ol	10.638	128.21	$C_8H_{16}O$	3391-86-4
trans-2-Undecen-1-ol	13.335	170.29	$C_{11}H_{22}O$	18409-17-7
1-Octanol	14.261	130.23	$C_8H_{18}O$	111-87-5
1-Decanol	16.568	158.28	$C_{10}H_{22}O$	112-30-1
1-Octanol, 2-butyl-	18.192	186.33	$C_{12}H_{26}O$	2/8/3913
2-Butyl-2,7-octadien-1-ol	25.186	154.25	$C_{12}H_{22}O$	14367-88-9
**Esters**				
Pentyl Acetate	7.917	130.18	$C_7H_{14}O_2$	628-63-7
Hexanoic acid, pentyl ester	22.351	186.31	$C_{11}H_{22}O_2$	818-29-3
**Organic Acids**				
17-Octadecynoic acid	17.997	280.46	$C_{18}H_{32}O_2$	59336-89-1
**Others**				
γ-Octalactone	21.271	142.2	$C_8H_{14}O_2$	104-50-7

Oxime-, methoxy-phenyl	8.347	137.15	C_8H_9NO	100-64-1
Cycloheptene, 1,2-dimethyl	12.587	110.19	C_9H_{16}	1192-37-6
2-Pentylfuran	11.077	138.21	$C_9H_{14}O$	3777-69-3
2-Methyldecalin	17.136	152.27	$C_{11}H_{2}0$	32918-38-6

Table S4. Interval of daily caloric request

Level of Activity	Male		Female	
Low	2200	2400	1800	2000
Moderate	2400	2800	2000	2200
High	2800	3000	2200	2400
